# Healthcare in the Dynamism of Metaverse After COVID-19: A Systematic Review of Literature

**DOI:** 10.7759/cureus.57554

**Published:** 2024-04-03

**Authors:** Mohit J Jain, Govinddas G Akbari, Yogesh N Umraniya, Shubham M Nagar, Nilkumar R Patel, Rushit H Shah, Chintankumar B Patel, Ravi P Undhad

**Affiliations:** 1 Orthopedic Surgery, Smt BK Shah Medical Institute and Research Centre and Dhiraj Hospital, Sumandeep Vidyapeeth, Vadodara, IND; 2 Anatomy, Gujarat Medical Education and Research Society (GMERS) Medical College, Morbi, IND; 3 Anatomy, Gujarat Medical Education and Research Society (GMERS) Medical College, Gandhinagar, IND

**Keywords:** virtual reality, telemedicine, metaverse, healthcare, augmented reality

## Abstract

The idea of the "metaverse" is a relatively recent technological development. The industries that are most supportive of these developments include finance, entertainment, and communication. In addition to these, the healthcare domain has been added to the list of domains that benefit from the metaverse recently. Within the metaverse, research is being conducted on a wide range of medical topics, including conferences and seminars, surgical simulators, awareness campaigns, research projects, and much more. The metaverse is a flexible and highly customizable virtual digital platform that can be configured to suit specific needs, making it an adaptable instrument for medical advancement. These domains, together with their benefits and drawbacks, are thoroughly covered in this review article, which raises the discussion of the need for medical productivity. These studies have undergone a minimum amount of research and experimentation, and the findings are fair from an investigative standpoint. This review article's major goal is to make a provocative remark about metaverse domains and how they have already been used and might be used as an essential operational tool in the field of medicine in the future. Consequently, the objective of the present study is to review the current literature on post-COVID-19 pandemic development that connected the metaverse with the prevention and treatment of diseases, medical education and training, and expansion of available functionalities in research settings.

## Introduction and background

Metaverse is a combination of two words "meta" and "universe." "Meta" means "beyond." So, the metaverse is a universe that is beyond the universe as we know it. It exists in the virtual realm but feels just as real. According to Lee LH et al., the metaverse is what happens when the real and virtual worlds band together to engage in social, commercial, and cultural endeavors that generate value [1]. Another way to describe it is as a three-dimensional virtual reality where everyday tasks and business dealings are carried out through virtual avatars or characters that represent various real-world characters. The idea of the "metaverse" first surfaced in the late 1990s. The term "metaverse" was originally used by Neal Stephenson in his 1992 book *Snow Crash*. It refers to the truth outside of reality in compilation. It is an amalgam of the terms "meta" and "universe," which stand for virtuality and transcendence, respectively. According to this concept, the "digitized earth" is a new universe expressed through digital media like cell phones and the internet. Much research and development turned the concept of the metaverse into a reality when it became a buzzword and gained extreme popularity. The idea of the metaverse was proposed and is currently the subject of much investigation in a number of development sectors. Tech behemoths like Facebook, Amazon, Microsoft, and many others are leading the way in important meta-developments, thus adding fuel to the fire [2,3].

**Figure 1:**
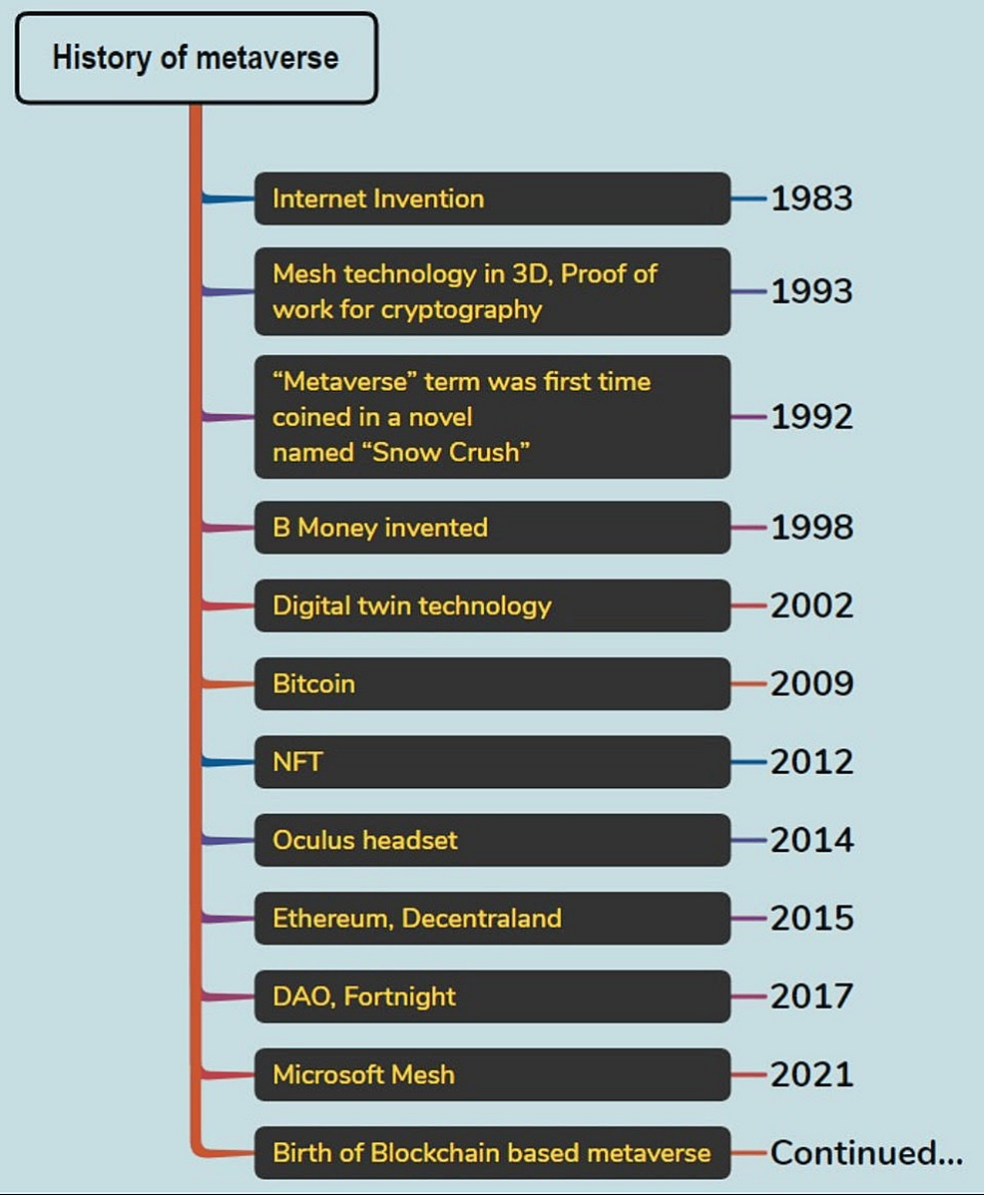
History and evolution of the current metaverse NFT: Non-fungible tokens; DAO: Decentralized autonomous organization This figure is an original work of the authors. Historical data for this figure have been provided (with written permission) by a metaverse company Parsh Technologies LLP (metaverse developers), Rajkot, Gujarat, India (https://parshtechnologies.com/).

Social media plays a significant role in the development of the metaverse. Facebook rebranded itself as Meta to reflect its goal of creating a metaverse out of the communication and information sphere [4]. Blockchain serves as the foundation for the metaverse, emphasizing its decentralized presence in the absence of a third-party supplier. As a result, the application of blockchain technology in the healthcare sector has become increasingly attractive with the rise of cryptocurrencies. Other potentially revolutionary modalities connected to the metaverse are non-fungible tokens or NFTs. As a token to claim ownership of a digital asset, NFT is a unique, non-transferable packet of data registered in the blockchain. A person can store their medical records and personal information for use in an emergency [5]. Figure 1 describes the historical evolution of the metaverse.

Configuration of the metaverse

**Figure 2:**
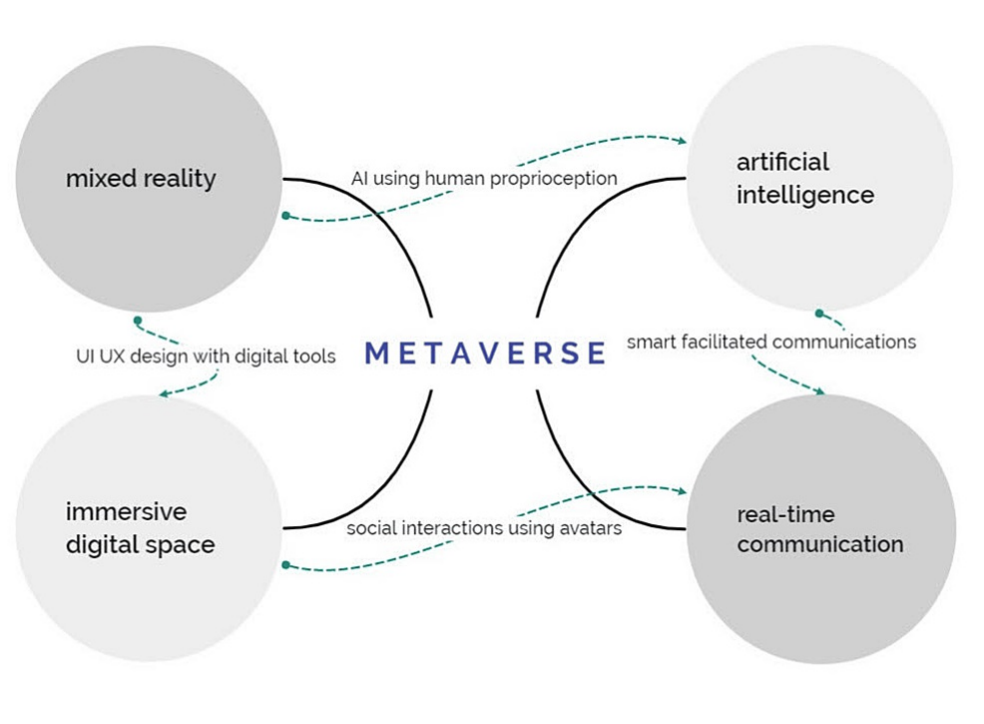
Individual and interconnected components of the current metaverse UI: User interface; UX: User eXperience This figure is an adaptation work (with written permission) from authors from the data/images provided by Parsh Technologies LLP (metaverse developers), Rajkot, Gujarat, India (https://parshtechnologies.com).

As stated in Jon Radoff's "Building the Metaverse," creating a sophisticated metaverse to take the place of the actual world is no simple task. The seven layers/elements required for such an accomplishment are (1) infrastructure, (2) human interface, (3) decentralization, (4) spatial computing, (5) creator economy, (6) discovery, and (7) experiences [6]. These layers are not strictly horizontal or vertical, instead, they intersect each other by a combination of multiple overlapping circles, thereby combining the potential of each sector. Also, there are four main versions of the metaverse: augmented reality, lifelogging, mirror worlds, and virtual worlds [7]. Figure 2 depicts individual and Interconnected components of the current metaverse.

**Figure 3:**
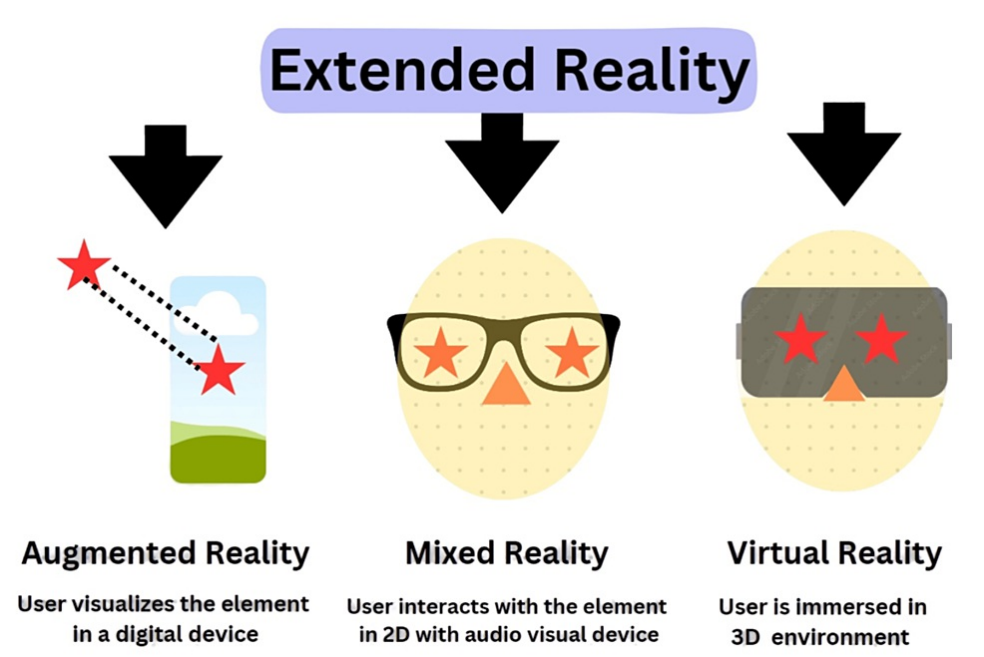
Components of extended reality This figure is an adaptation work (with written permission) from authors from the data/images provided by Parsh Technologies LLP (metaverse developers), Rajkot, Gujarat, India (https://parshtechnologies.com).

The current metaverse is a platform that facilitates social and commercial interactions between the virtual and real worlds by fusing many technologies, including artificial intelligence (AI), mixed reality (MR), immersive digital space, and real-time communication. The metaverse needs to satisfy the three Ps: presence, persistence, and portability, to provide a fully immersive experience. Virtual reality (VR) and augmented reality (AR) are both included in MR (Figure 3).

Both let people utilize a smartphone for a less immersive experience or a head-mounted gadget to fully immerse themselves in digital information. A fully digital universe created to be apart from the real world is called VR. VR headsets enlarge the user's range of view and need to include a feature that lets them interact with their virtual world. A computer overlay superimposed over the actual world is known as augmented reality. These transparent headsets project digital content on top of the user's actual surroundings, enabling a clear vision of it. For these headsets to project digital content at the proper location and scale, they need to be able to map the surrounding world in three dimensions. Extended reality combines everything-essentially akin to the metaverse [8].

## Review

Literature search strategy

**Figure 4:**
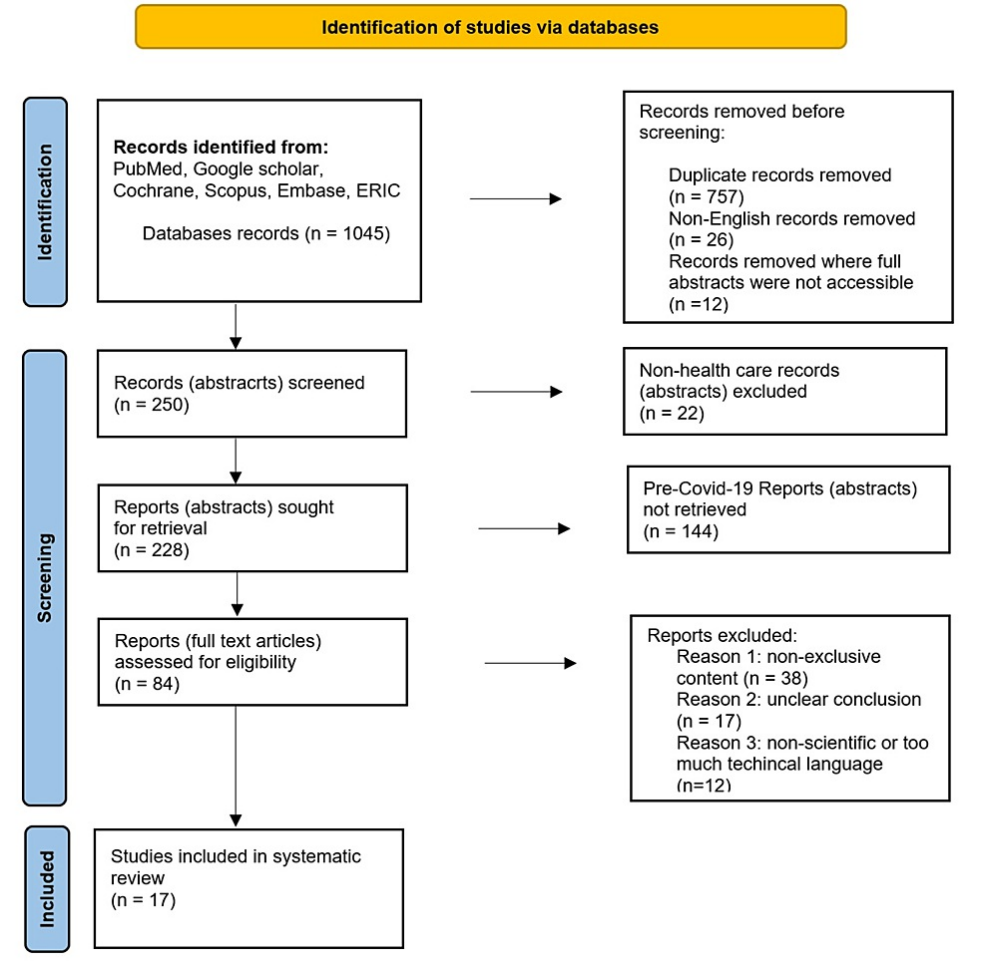
Study design methodology PRISMA flowchart PRISMA: Preferred Reporting Items for Systematic Reviews and Meta-Analyses

To address the research question (what are potential new developments of the metaverse in healthcare?) and to review available post-COVID-19 pandemic literature (April 2020-January 2024), we navigated through six databases (PubMed, Google Scholar, ERIC {Educational Resource Information Center}, Scopus, Embase, and Cochrane) by searching the Mesh term in combination as "(metaverse) AND (healthcare OR medicine)". Using the scientific writing tool Mendeley (with Windows software, Microsoft Word extension, and Google Chrome extension), we gathered all literature into its e-library and classified them according to set inclusion and exclusion criteria as mentioned in PRISMA (Preferred Reporting Items for Systematic Reviews and Meta-Analyses) checklist in flowchart (Figure 4).

Inclusion and exclusion criteria

We included all the articles where full abstracts were available discussing the development in clinical healthcare (or its components like education and research) via the metaverse (or its components like VR, AR, blockchain, etc.). While navigating through available literature, we found 1045 use cases for metaverse in healthcare. We excluded duplicate articles, articles not related to healthcare, non-English articles, and pre-pandemic articles by reviewing abstracts. Lastly, the first two authors performed qualitative analysis for innovative content, ease of language, and clarity of conclusion for inclusion of the studies.

Data extraction and analysis

We categorized the results into three categories: (1) health prevention and the treatment of clinical conditions, (2) education and/or training, and (3) research. We included studies from 17 studies for systematic review. Despite overlapping, broadly speaking, seven were healthcare, seven were education, and three were research-based category studies. Although all of them were more or less on the use cases of the metaverse, their conceptual inclusions were also classified as the Internet of Medical Things (IoMT), VR, AR, MR, blockchain, and digital twinning.

Through algorithm training, AI can create real-time disease information according to the patient’s disease course development and provide timely feedback on the doctor’s diagnosis and treatment information. This will preserve the information ecology of the material and significantly increase the efficiency of content development. SLIDES (Second Life Impacts Diabetes Education and Support) is a virtual environment designed for diabetes self-management that was created through another study [9]. People could access virtual structures on SLIDES as avatars, including restaurants, bookstores, and more. The avatars could engage with a virtual grocery shop that offered nutritional advice, a gym where users could exercise (which they could imitate at home), and classes where they could learn from diabetes instructors and other participants who also had diabetes. Sessions take place in actual clinics and classrooms and are frequently removed from participants' everyday lives, which could help users overcome obstacles.

Internet of Medical Things (IoMT)

The metaverse has entered people's horizons through VR, digital twinning, the IoMT, blockchain technology, etc. [10]. An efficient system for producing material is essential to the establishment of the metaverse information ecology. 3D scanning, dynamic point cloud technology, and voxel to compensate for the shortcomings of the grid model allow for the rapid creation of digital assets. Together with metaverse extended reality, real-time rendering engines and hardware rendering capabilities progress along with cloud rendering technology.

To realize the "simplification of complex problems, digitalization of simple problems, programming of digital problems, and systematization of programming problems," Yang D et al. proposed expanding the concept by adding intelligent processing to the holographic emulation, quality control to the virtuality-reality integration, and human-computer integration to the virtuality-reality interconnection [11]. This approach can overcome the obstacle that internet-based healthcare and telemedicine platforms hardly play an active role in county hospitals, especially those in rural villages and towns. Moreover, it could facilitate graded diagnosis and treatment, and contribute to transforming the current handicraft workshop model, which varies between doctors and hospitals with uneven levels, into a modern assembly-line model that meets national and even international standards.

The medical field has already established a strong technological base through the use of a wide range of sensors that apply force, gas, photosensitive, radiation, ultrasound, electrocardiography (ECG), computed tomography (CT), positron emission tomography (PET), spirometers, and pulse oximeters. Biochemical examinations are also used to test liver and kidney function. These technologies allow us to produce a comprehensive picture of the state of health, sub-health, or disease as well as track physiological, pathophysiological, and biochemical changes in the body at all times and in all locations [11]. As a result, via virtuality-reality interconnection, medical professionals and patients can practice metaverse medicine by entering the metaverse with their digital twins. Patients and physicians will only participate in the metaverse if it produces an immersive experience making it impossible for them to tell the difference between actual and virtual worlds.

Medical training

According to the results of the study by Alawadhi M et al., learners' impressions of using the metaverse and their degrees of inventiveness are closely related [12]. Users of the metaverse system expressed appreciation for one of the innovations that will significantly alter medical education. In the modern world, innovative technology makes a variety of instructional approaches possible. The web of the internet may be replaced by blockchain technology in the future, which will also bring more innovation and revolutionize teaching and learning.

Kim Y et al. discovered that mentees' self-efficacy in making professional decisions increased significantly after participating in a metaverse-based career mentoring program, as compared to their baseline level [13]. Three main themes emerged from the focus group interviews between mentors and mentees: (1) being honest and forthright in communication, (2) accepting realistic communication and program functions, and (3) anticipating an even more streamlined program. The self-efficacy of nursing students in making career decisions can be enhanced by a metaverse-based career coaching program.

A study by Chua HW et al. maps the trends of changes in the use of metaverse in education and examines its perceived usefulness and perceived ease of use from 2008 to 2022 [14]. The research findings are concluded as follows: (1) the trend of change from a single platform or software of the metaverse to a more diverse combination of software and devices among the types of metaverse in education, and (2) the importance of perceived usefulness and perceived ease of use in the acceptance and rejection of the use of metaverse in education.

A gastroenterology training study done by Zhang C et al. found that the current generation of endoscopic simulators only offers basic training for endoscopists and is unable to replicate more complex surgical procedures, such as endoscopic mucosal resection (EMR), endoscopic submucosal dissection (ESD), or other treatment procedures, which signiﬁcantly restricts the development of endoscopists [15]. However, the metaverse can eventually oﬀer us all the scenarios that need to mimic, allowing us to overcome the constraints of models and technology because it is a virtual world that is entirely separate from the real world. Only endoscopists can be given access to metaverse devices, can utilize VR helmets and tactile gloves, and have ﬁnished advanced endoscopic training. Additionally, AI can be used to search for professional training videos in cloud databases and master the close-up endoscope treatment operation abilities in the metaverse. As a result, the risk of surgical failure can be prevented in addition to gaining extensive endoscopic expertise.

Management of mental disorders

A number of commissions and committees have suggested using AR and VR in the past few years to help autistic people develop new interpersonal skills and talents. VR can become the perfect tool for intervention and rehabilitation by providing a more welcoming and realistic environment. Certain medical or mental illnesses might make it difficult for a person to take care of oneself or control their behavior. They can also make a person feel uncomfortable in social circumstances. These subjects must improve social interaction skills because of this, especially in adolescence. VR technology provides a safe, regulated environment in which therapists can guide patients through incremental, one-on-one therapies. It has been shown that behavioral therapies mediated by VR technology improve motivation, attention, and social skills. People with ASD (autism spectrum disorder) may avoid social situations in part due to social anxiety brought on by failed attempts at interactions [16].

Digital twins and simulations

Digital twin technology offers truly remarkable outcomes for the healthcare industry. Imagine being able to create a virtual representation of a patient equipped with real-time data from medical devices like heart monitors. It allows healthcare professionals to closely monitor patients’ vital signs and identify potential issues. They can prevent costly medical interventions and improve patient outcomes by taking proactive steps. Additionally, one can create a digital twin of an MRI machine or other medical equipment, so healthcare professionals can closely monitor its performance in real-time. If any patterns or trends emerge to indicate a problem with the machine, they can take preventative measures to avoid costly downtime and improve the overall performance of the equipment [17]. Another technology that mimics reality is simulation. For example, soldering and welding simulations, shooting simulations, or medical procedures simulations, this technology can be utilized to create an interactive environment. A sort of technology known as "intimate technology" allows the real world to be represented by an avatar.

Although simulations and digital twins both utilize digital models to replicate a system’s various processes, a digital twin is a virtual environment, which makes it considerably richer for study. The difference between a digital twin and a simulation is largely a matter of scale: while a simulation typically studies one process, a digital twin can run any number of useful simulations to study multiple processes [18]. Patient monitoring can continue thanks to the integration of digital twins with real-time data from wearables, IoMT devices, and remote monitoring systems. Digital twins monitor physiological parameters, vital signs, and other health-related data in real time, enabling the detection of anomalies or early indicators of decline. This makes it possible for medical professionals to take preventative measures, avoid complications, and enhance treatment regimens. Patients can take a more active role in managing their health by having access to their digital twin data, which includes individualized health insights, treatment plans, and progress tracking [19].

Augmented reality (AR)

Liu K et al. developed an AR navigation technique for recontouring craniofacial fibrous dysplasia to overcome the aforementioned limitations [20]. The technique combines virtually planned and real images into one prospect, enabling the viewing of the imaging, surgical field, and additional information such as the location of the surgical instrument in real-time and improving the visualization of every possible narrow space. Configuration of the proposed AR navigation system: (1) digital reference frame fixed to the patient’s craniofacial skeleton, (2) surgical drill with clamped digital reference frame; (3) optical tracking system and workstation; (4) head-mounted display, and (5) 3D virtual planning and the position of surgical drill. They prepared the application of an AR navigation system in five consecutive patients diagnosed with craniofacial fibrous dysplasia undergoing surgical recontouring. Accordingly, they investigated and found a positive correlation in the feasibility of using the AR system to enhance the surgical outcome measured by increasing accuracy and decreasing errors.

Elmi-Terander A et al. compared screw placement accuracy and clinical aspects between Augmented Reality Surgical Navigation (ARSN) and free-hand (FH) technique [21]. Twenty patients underwent spine surgery with screw placement using ARSN and were matched retrospectively to a cohort of 20 FH technique cases for comparison. All ARSN and FH cases were performed by the same surgeon. Matching was based on clinical diagnosis and similar proportions of screws placed in the thoracic and lumbosacral vertebrae in both groups. This matched-control study demonstrated that ARSN provided higher screw placement accuracy compared to FH. Use cases of such techniques are also possible in human education for the microscopic dissection of human cadavers [22].

Kim D et al. found that AR smart glass applications offer the highest potential for service innovation in the healthcare sector [23]. Smart glass prototype supports healthcare professionals during wound treatment by allowing them to document procedures hands-free while they perform them. Furthermore, they investigated the use of audio-based and physical interaction with smart glasses in a within-subjects design experiment.

Rojas-Munoz E et al. evaluated the system for telementoring with augmented reality (STAR), a portable and self-contained telementoring platform based on an augmented reality head-mounted display (ARHMD) [24]. The system was designed to assist in austere scenarios: a stabilized ﬁrst-person view of the operating ﬁeld is sent to a remote expert, who creates surgical instructions that a local ﬁrst responder wearing the ARHMD can visualize as three-dimensional models projected onto the patient’s body. The hypothesis evaluated whether remote guidance with STAR could lead to performing a surgical procedure better, as opposed to remote audio-only guidance. Remote expert surgeons guided ﬁrst responders through training cricothyroidotomies in a simulated austere scenario, and on-site surgeons evaluated the participants using standardized evaluation tools. The evaluation took into account the execution time and technique of particular cricothyroidotomy phases. In addition, the analyses took into account the years of first responder experience and cricothyroidotomy experience of the subjects. Using STAR was linked to greater overall performance as well as higher procedural and non-procedural ratings, according to a linear mixed model study. Furthermore, a binary logistic regression analysis demonstrated that the use of STAR was linked to safer and more effective cricothyroidotomy procedures. According to them, this work was a first step toward the implementation of ARHMDs to transmit clinical experience remotely in harsh circumstances, since it demonstrated how remote mentors can use STAR to guide and educate first responders about surgery.

Zimmer-Biomet, the medical equipment manufacturer, has introduced OptiVuTM software which gives physicians and patients access to holographic images [25]. Microsoft HoloLens is used to combine the real and virtual worlds. Patients will be able to duplicate realistic consultations, individualized care, and therapy with the help of data interconnection. Therefore, by bridging the gap between the patient and the doctor, the metaverse has extreme potential to positively alter the healthcare sector [26].

Variety of multiple use cases

The first attempt to provide a thorough analysis and a wide variety of the most recent metaverse advancements in the healthcare sector is presented by Bansal G et al. and covers seven domains: telemedicine, clinical care, education, mental health, physical fitness, veterinary, and pharmaceuticals [27]. They went over metaverse applications and had in-depth discussions on the technological problems and potential fixes in each area to aid in the creation of a long-lasting, self-sufficient, and future-proof medical healthcare system solution. They draw attention to the obstacles that need to be overcome before the healthcare sector can completely embrace the metaverse. In our view, this study was one of the most extensively organized collections of use cases of metaverse in healthcare.

Genetic counseling

Yoo B et al. performed group genetic counseling on a group of patients and found that a metaverse platform could be an alternative service-delivery model for group genetic counseling [28]. This is different than previous studies in the past comparing telephonic versus in-person genetic counseling. This indicates that metaverse platforms can be expected to be effective in achieving such an objective.

Deep learning with metaverse platforms

A blockchain-based metaverse platform is built and implemented to house a digital twin of cervical vertebral maturation (CVM), which is a crucial component in many types of dental surgery, as demonstrated by Moztarzadeh O et al. [29]. An automated diagnosis procedure for the forthcoming CVM images in the suggested platform has been developed using a deep-learning (DL) technique. The study used DL-based computer vision as a real-time measuring technique to eliminate the need for extra sensors in the suggested digital twin. Additionally, a thorough conceptual framework based on MobileNetV2 technology was put into practice for producing digital twins of CVM within a blockchain ecosystem, demonstrating the compatibility and application of the previously stated approach. Furthermore, this study also demonstrated how dental problems can be digitally twined and developed with the least amount of hardware infrastructure, saving patients' costs associated with diagnosis and treatment.

Teleconsultations

The metaverse allows people to connect through virtual reality, unlike Second Life, an online multimedia platform that was launched in 2003 that allows users to create an avatar and interact with others in a virtual world, which is only two-dimensional [30]. By using it, patients can virtually meet their doctor to discuss their case or even have their data analyzed, which is far more realistic than meeting in person or using a telemedicine app or video chat. A patient might visit a virtual pharmacy to pick up their prescription medication, chat with their cardiologist via a digital avatar, and even have their appointment in a virtual office (or VR app). After that, they can get together with other patient communities to discuss recent advancements related to their conditions and to report, in avatar form, the status of their therapy.

The details of the 17 studies analyzed are listed in Table 1.

**Table 1:** Brief summary of studies included for systematic analysis VR: Virtual reality, DT: Digital twinning, IoMT: Internet of Medical Things, AR: Augmented reality, AI: Artificial intelligence, UAE: United Arab Emirates

Sr No.	Author	Journal	Year	Brief title	Conceptual inclusions	Category
1	Sun M et al. [10]	Clinical eHealth	December 2022	The metaverse in current digital medicine	VR, DT, IoMT, blockchain	Healthcare
2	Yang D et al. [11]	Clinical eHealth	December 2022	Expert consensus on the metaverse in medicine	IoMT, IoMT, AR, VR	Healthcare
3	Alawadhi M et al. [12]	South Eastern European Journal of Public Health	May 2022	Factors affecting medical students’ acceptance of the metaverse system in medical training in the UAE	Metaverse	Education
4	Kim Y et al. [13]	BMC Nursing	May 2023	Effects of metaverse-based career mentoring for nursing students: a mixed methods study	Metaverse	Education
5	Chua HW et al. [14]	Journal of Computer Education	May 2023	A systematic literature review of the acceptability of the use of the metaverse in education	Metaverse	Education
6	Zhang C et al. [15]	Frontiers in Medicine (Lausanne)	August 2022	Gastroenterology in the metaverse: the dawn of a new era?	Metaverse, AI	Healthcare
7	Cerasa A et al. [16]	Heliyon	March 2023	The promise of the metaverse in mental health: the new era of MEDverse	Metaverse, AR	Healthcare
8	Vallee A [19]	Frontiers in Digitial Health	September 2023	Digital twin for healthcare systems	DT	Healthcare
9	Liu K et al. [20]	Scientific Reports	May 2021	Augmented reality navigation for recontouring surgery of craniofacial fibrous dysplasia	AR	Research
10	Elmi-Terander A et al. [21]	Scientific Reports	Jan 2020	Augmented reality navigation with intraoperative 3D imaging vs fluoroscopy-assisted free-hand surgery for spine fixation surgery: a matched-control study comparing accuracy.	AR	Research
11	Zammit C et al. [22]	Clinical Anatomy	September 2022	Augmented reality for teaching anatomy.	AR	Education
12	Kim D et al. [23]	Applied Sciences	May 2021	Applications of smart glasses in applied sciences: a systematic review	MR	Healthcare
13	Rojas-Munoz E et al. [24]	Surgery	April 2020	STAR: A head-mounted display to improve surgical coaching and confidence in remote areas	AR	Education
14	Bansal G et al. [27]	IEEE Access	Nov. 2022	A survey on current metaverse applications in healthcare	Metaverse, AR, VR	Healthcare
15	Yoo B et al. [28]	Annals of Laboratory Medicine	Jan. 2024	Evaluation of group genetic counseling sessions via a metaverse-based application	Metaverse	Education
16	Moztarzadeh O et al. [29]	Diagnostics	April 2023	Metaverse and medical diagnosis: a blockchain-based digital twinning approach based on MobileNetV2 algorithm for cervical vertebral maturation	Metaverse, DT, blockchain	Research
17	Boulos MN et al. [30]	Health Information and Libraries Journal	April 2023	An overview of the potential of 3-D virtual worlds in medical and health education	VR	Education

Limitations

One of the limitations of our study is the subjective/observer bias. Besides, our wide exclusion criteria may have produced some sampling bias. However, the major strength of our study is that it addresses the recent trend on this topic especially post-COVID-19 since we are expecting a major boost in the concept in the near future. While exploring all the studies, we found an overwhelming result if we combined everything that existed before the emergence of the notion of metaverse as such. Because technologies like VR, AR, extended reality (XR), and AI were already in existence, the rise of blockchain technology primarily led by Bitcoin, the boom produced by the cryptocurrency market aroused the world to peep into the power of this potential technology, and it gave birth to the metaverse. Following the same, extreme corporate interest taken by the world’s giant companies added fuel to the fire, and the metaverse combined everything in the name of technology that existed into one cocktail. New developments are made daily to enhance metaverse modalities' output. Despite all of the technological progress that has been made, there is still room for it to grow in all directions. Identical characteristics are evident in the field of medicine. The benefits of the metaverse outweigh the drawbacks when all the information about it is considered. Even the drawbacks, however, have room for improvement and can be resolved with additional technology advancement, the implementation of user ethics, rules and oversight, and judicial usage.

## Conclusions

The introduction and application of metaverse in key medical domains will improve the provision of healthcare services. While this is still in the experimental stage, it is acceptable to ignore the metaverse's cost-effectiveness for the time being. Problems such as global availability and cost efficiency can also be resolved if sufficient study has been conducted. A dynamic and long-lasting instrument for the general advancement of the medical industry is the metaverse. This reform has the potential to help not only the medical industry but also its downstream industries, including finance, human resource development, and pharmaceuticals. It will take a great deal of investigation and testing to refine the technology. and it is crucial to use it when discussing ethical issues. It still begs the question of whether it truly is a technology that will transform to a high level of usability in various fields, and if yes, to what extent.

As discussed in telemedicine use cases, compared to existing telemedical visits that take place through a flat screen or online patient forums, metaverse presents a chance to improve the user experience. Given the increasing frequency of these visits, giving patients access to an improved interface may encourage medical professionals to provide their services across the metaverse. Furthermore, because this "new internet" appears to be highly interconnected, a patient's digital avatar may contain any digital data, including digital medical records gathered from wearables and direct-to-consumer services, which they might exchange with their doctor in the healthcare metaverse. Healthcare providers could collaborate on more complex cases and offer help to one another. Surgeons could examine the same structures in three dimensions utilizing virtual reality for pre-operative planning, based on echocardiography data. With fast 5G or perhaps even faster 6G internet, professional metaverse operations in a virtual environment are going to be the future of healthcare in prevention as well as management including interventional or surgical treatment.
